# A Data-Driven Method to Reduce the Impact of Region Size on Degree Metrics in Voxel-Wise Functional Brain Networks

**DOI:** 10.3389/fneur.2014.00199

**Published:** 2014-10-13

**Authors:** Cirong Liu, Xiaoguang Tian

**Affiliations:** ^1^Queensland Brain Institute, The University of Queensland, St Lucia, QLD, Australia; ^2^Werner Reichardt Centre for Integrative Neuroscience, Tübingen, Germany

**Keywords:** degree, functional hubs, resting state fMRI, brain network, region growing

## Abstract

Degree, which is the number of connections incident upon a node, measures the relative importance of the node within a network. By computing degree metrics in voxel-wise functional brain networks, many studies performed high-resolution mapping of brain network hubs using resting-state functional magnetic resonance imaging. Despite its extensive applications, defining nodes as voxels without considering the different sizes of brain regions may result in a network where the degree cannot accurately represent the importance of nodes. In this study, we designed a data-driven method to reduce this impact of the region size in degree metrics by (1) disregarding all self-connections among voxels within the same region and (2) regulating connections from voxels of other regions by the sizes of those regions. The modified method that we proposed allowed direct evaluation of the impact of the region size, showing that traditional degree metrics overestimated the degree of previous identified hubs in humans, including the visual cortex, precuneus/posterior cingulate cortex, and posterior parietal cortex, and underestimated the degree of regions including the insular cortex, anterior cingulate cortex, parahippocampus, sensory and motor cortex, and supplementary motor area. However, the locations of prominent hubs were stable even after correcting the impact. These findings were robust under different connectivity thresholds, degree metrics, data-preprocessing procedures, and datasets. In addition, our modified method improved test–retest reliability of degree metrics as well as the sensitivity in group-statistic comparisons. As a promising new tool, our method may reveal network properties that better represent true brain architecture without compromising its data-driven advantage.

## Introduction

The brain can be conceptualized as a complex network with a few highly connected regions that support neural communication (1). Over the last two decades, techniques used to characterize the brain network have rapidly improved from post-mortem tracing techniques in animals (2–4) to *in vivo* brain imaging methods in humans (5, 6). In particular, researchers have begun to use the resting-state functional magnetic resonance imaging (rs-fMRI) to characterize functional brain network at a voxel-wise spatial resolution (7). In the voxel-wise network, each voxel corresponds to a node, and the edge between two nodes is defined by functional connectivity, which is the temporal correlation of spontaneous fluctuations of blood oxygenation level-dependent (BOLD) signals between any two nodes (8). Degree, which is the number of connections incident upon a node, can measure the relative importance of a node within a network (9). Using degree metrics, previous studies identified “functional hubs” that were highly connected nodes in brain networks (7, 10). This voxel-wise degree-based method is data-driven, having no need to define any regions of interest, and has extensive applications in studying brain network architectures and brain disorders (10–20).

The degree-based method has recently been argued that it may not accurately represent the importance of nodes in the voxel-wise functional brain networks (21, 22). Different sizes of functional regions, which consist of spatially adjacent voxels that show coherent spontaneous BOLD signal oscillations, may affect the voxel-wise framework. In this framework, a voxel’s degree scales with (1) the size of the functional region to which it belongs and (2) the sizes of the functional regions to which it connects. Thus, the hubs detected by degree metrics tend to be functional regions/systems that comprise the largest number of voxels. However, degree is still an indispensable measure in brain network analysis, considering the fact that the traditional degree-based method has extensive applications, beyond just the search for hubs. It is therefore imperative to consider methods that resolve the drawbacks of the traditional degree-based method.

**Figure 1:**
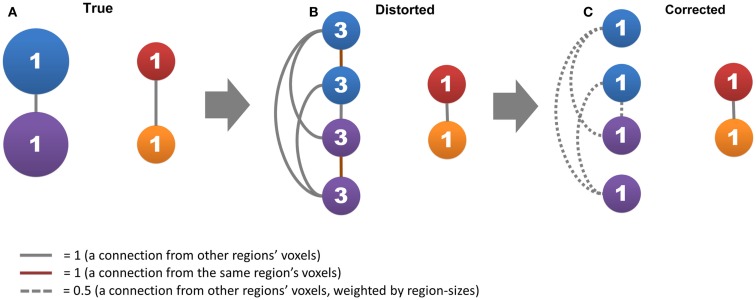
**The region-size impact and its correction strategy are shown**. Circles represent nodes, where the network has two connected large functional regions that each consists of two voxels (blue and purple circles) and two connected small functional regions that each consists of one voxel (red and orange circles). Lines represent connections, where gray lines represent connections from other functional regions, red lines represent self-connections, and dashed lines represent connections divided by the size of the functional regions. The number in a circle represents the degree of centrality. **(A,B)** show how the voxel’s degree scales with functional region size, and **(C)** shows the effective strategy to correct this impact.

In the light of the vacancy, we propose a modified version of degree-based method that can reduce the impact of region size but retaining its data-driven advantage. Briefly, this method is achieved by (1) disregarding self-connections among voxels within the same region and (2) regulating connections from voxels of other regions based on the sizes of those regions (Figure 1). The prerequisite of this strategy is to define the sizes of functional regions. In general, one way to approach this problem is to refer to anatomical brain atlases or predefined brain parcellations, but these are either inaccurate or inconsistent (23–26). Moreover, there is no consensus on how many functional regions the brain contains. Instead, we use a modified region-growing method to estimate the size of the functional region to which each voxel may belong, and then perform the above strategy. Our modified method inherits the data-driven advantage of traditional degree-based method and allows direct evaluation of this impact of region size. In addition, we also evaluate the test–retest reliability and other factors that may influence degree metrics, including global signal regression (GSR), head motions, network types (unweighted and weighted), and connectivity thresholds, as well as testing the sensitivity in group-statistic comparisons. The comprehensive assessment will prove the reliability of our method as a new tool for the degree-based analysis.

## Materials and Methods

### Subjects and data acquisition

Public dataset, NewYork_Test-Retest_Reliability (NY_TRT)^1^ of the 1,000 Functional Connectomes Project, was used (27). The NY_TRT dataset consists of 6.5-min scans acquired from 25 healthy subjects (10 males and 15 females) on a 3-T Siemens Allegra scanner using an echo-planar imaging (EPI) sequence: time repetition (TR) = 2000 ms; time echo (TE) = 25 ms; flip angle = 90°; 39 slices, matrix = 64 × 64, field of view (FOV) = 192 mm; acquisition voxel size = 3 mm × 3 mm × 3 mm. A high-resolution T1-weighted anatomical image using magnetization prepared gradient echo (TR = 2500 ms; TE = 4.35 ms; flip angle = 8°; 176 slices, FOV = 256 mm) was also obtained for spatial normalization. There were three scans in the NY_TRT dataset. Scan 1 was collected 5–16 months (mean ± SD = 11 ± 4) before scans 2 and 3, which were collected in a single session 45 min apart. In order to keep the largest head image, six subjects who did not have half the cerebellum or lost voxels in the top slice of the brain in any of the three scans were excluded from the current study.

Public dataset, NYU Institute for Pediatric Neuroscience – Cocaine (NY_Cocaine) of the 1,000 Functional Connectomes Project was used to test the sensitivity of degree metrics in group-statistic comparisons. The NY_Cocaine dataset comprises 24 healthy subjects and 29 cocaine-dependent subjects. One 6-min resting state scan was acquired for each subject, using an EPI sequence on a 3-T Siemens Allegra scanner (180 time points; TR = 2000 ms; flip angle = 90°; 33 slices; voxel size = 3 mm× 3 mm × 4 mm). Detailed demographic information and data acquisition protocols have been described in a previous publication (28).

### Data preprocessing

Data preprocessing of the NY_TRT dataset was performed using the Data Processing Assistant for Resting-State fMRI (29), which was based on the Statistical Parametric Mapping (SPM8)^2^ and the Resting-State fMRI Data Analysis Toolkit (30). Functional images were slice time corrected, after which the time series of each subject was realigned using a rigid body linear transformation with a two-pass procedure (registered to the first image and then registered to the mean of the images after the first realignment). Individual structural image was co-registered to the mean functional image by a rigid body linear transformation without re-sampling. The transformed structural images were segmented into gray matter, white matter, and cerebrospinal fluid (31), and transformations from individual native space to MNI space were computed using the Diffeomorphic Anatomical Registration Through Exponentiated Lie algebra (DARTEL) tool (32). Functional images were normalized into MNI space with 4 mm^3^ cubic voxels by using transformation information acquired from DARTEL, and then were denoised by regressing out linear and quadratic trends, six motion parameters, white matter signals, and cerebospinal fluid signals. Finally, temporal filtering was applied to functional images with a band-pass filter (0.01–0.1 Hz). To exclude artificial correlations from the non-gray matter voxels and reduce computational cost, we restricted our analysis to a predefined gray matter mask with gray matter tissue probability >20% (20). No smoothing was performed in the preprocessing as it would blur the data, introduce artificial local correlations, and affect the degree analysis (7, 20). Data preprocessing of the NY_Cocaine dataset was similar, except that the NY_Cocaine dataset had been provided in the MNI space. Thus, the spatial normalization was skipped in its preprocessing.

We also performed different preprocessing procedures to examine potential confounding factors. As *GSR* affected correlation distribution (11, 33), we included global signals as regressors to evaluate the *GSR* effect. Besides *GSR*, recent studies have suggested that head motion affects functional connectivity (34, 35), thus we also used Friston-24 model to reduce the influence of head movement. Results with *GSR* and Friston-24 were similar as the results without them.

### Proposed method for computing degree metrics

**Figure 2:**
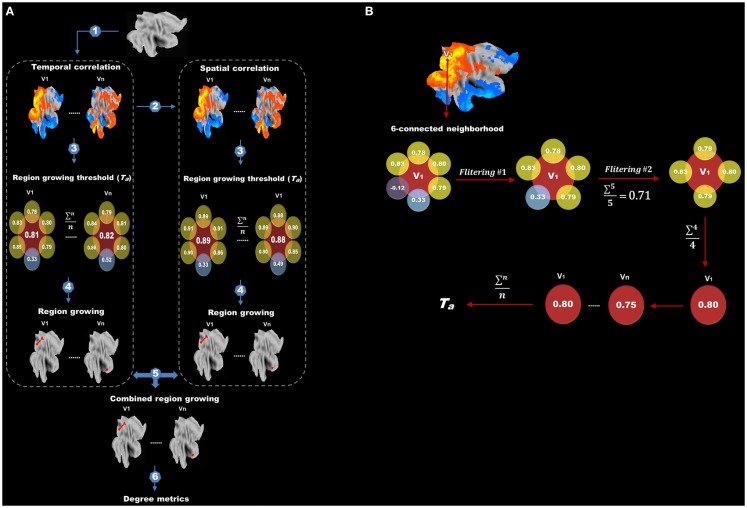
**Flow chart of protocols after data preprocessing is shown**. **(A)** Protocols after data preprocessing include (1) temporal correlation analysis, (2) spatial correlation analysis, (3) determining adaptive thresholds for region growing, (4) region growing, (5) combining region-growing results and (6) degree analysis. **(B)** The flow chart for finding the adaptive threshold. Correlations between the target voxel (*V*_1_) and its six adjacent voxels are extracted. Negative and extreme low correlations are first excluded (first filtering) and the remainders are averaged. Any adjacent voxels with a correlation lower than the average are further discarded (second filtering). The correlation values between *V*_1_ and its surviving adjacent voxels are again averaged to obtain a threshold for *V*_1_. This procedure is repeated for every voxel in the brain, and the final threshold (*T_a_*) for region growing is calculated by averaging the thresholds of all voxels.

After data preprocessing, we performed our modified method for degree analysis (the flow chart is shown in Figure 2A). The following steps were carried out on each subject: (1) temporal correlation analysis; (2) spatial correlation analysis; (3) determining adaptive thresholds for region growing; (4) region growing based on temporal and spatial correlation, respectively; (5) combining region-growing results from temporal and spatial correlation to estimate the sizes of functional regions; and (6) degree analysis. In what follows, we detailed the methods associated with each of these steps.

#### Temporal correlation analysis

The temporal correlation between a pair of voxels was represented by the Pearson correlation coefficient of their preprocessed time series (8). The temporal correlation map of each voxel involved its temporal correlations with all other voxels of the brain.

#### Spatial correlation analysis

The spatial correlation between a pair of voxels was calculated by the Pearson correlation coefficient of their whole-brain temporal correlation maps. The spatial correlation map of each voxel involved its spatial correlations with all other voxels of the brain. As a spatial correlation was derived from temporal correlation, they were similar but not identical, reflecting different functional connectivity features. The information used to calculate the temporal correlation was derived from preprocessed time series, whereas the information for spatial correlation involved whole-brain functional connectivity patterns. Due to voxels in the same functional region should not only have high temporal correlation with each other but also similar whole-brain functional connectivity patterns, we performed region growing on both temporal and spatial functional connectivity maps for a better estimation of region sizes.

#### Adaptive threshold for region growing

We tried to find an adaptive (data-driven) threshold (*T_a_*) for region growing based on the assumption that the correlation between a target voxel and its nearest neighbors (six spatially adjacent voxels) was higher than that with other distant voxels, thus the target voxel and its nearest neighbors were more likely to be in the same functional region. As illustrated in Figure 2B, correlation values between the target voxel (*V*_1_) and its six spatially adjacent voxels were computed. Negative correlations and extremely low correlations, which are lower than the mean positive correlation of the whole correlation matrix were first excluded (first filtering) and then any adjacent voxels with a correlation value lower than the average value of the remaining voxels was further discarded (second filtering). This two-step filtering can exclude negative and low correlations and keep high correlations for a better estimation of *T_a_*. Finally, the correlation values between this target voxel *V*_1_ and its surviving adjacent voxels were averaged to obtain a threshold for this target voxel *V*_1_. This procedure was carried out for every voxel in the brain, and the final threshold (*T_a_*) for region growing was calculated by averaging the thresholds of all voxels.

Repeated measures ANOVA followed by the Bonferroni multiple comparison test was conducted to evaluate inter-session reliability of *T_a_* among the three scans. A paired two-sample *t*-test was also conducted between thresholds from data with and without *GSR* to evaluate whether *T_a_* was adaptive to different preprocess strategies.

#### Region growing

Region growing was performed on every voxel based on the threshold *T_a_* obtaining from above step. The region-growing algorithm was a modified version adapted from previous studies (12, 15) and can be summarized as following:

$$C_{i,t+1}=\left\{\begin{array}{cc} j\in C_{i,t}\; & \mathrm{if}r_{t}\left(i,j\right)\geq T_{a}\\ j\notin C_{i,t}\; & \mathrm{if}r_{t}\left(i,j\right)<T_{a}\\ \; \end{array}\right.$$

where, *i* ∈ brain voxels (a starting voxel of region growing); *j* ∈ 26 spatial adjacent voxels of *C_i_*; “*t*” represents the *t*-th iteration; *r_t_*(*i, j*) is the correlation between voxels *i* and *j* at the *t*-th iteration; *T_a_* is adaptive threshold.

Taking a voxel (*V*_0_) as example, voxels within the same functional region as *V*_0_ were defined as cluster *C*_0_. Before the first round of region growing, only *V*_0_ was included in *C*_0_, after which any adjacent voxels of *C*_0_ was added to *C*_0_ only if its correlation with *V*_0_ was larger than *T_a_*; otherwise *C*_0_ remained unchanged. This procedure was repeated for all voxels adjacent to *C*_0_ iteratively until no new voxels could be added to *C*_0_.

#### Combining region growing and its evaluation

The procedure of region growing was implemented on both temporal correlation maps and spatial correlation maps, resulting in two clusters for each voxel. A paired two-sample *t*-test was used to test the difference between temporal and spatial region growing, and the intra-class correlation coefficient (*ICC*) (36) was used to examine the similarity between them. Finally, two clusters were intersected to obtain the combined region-growing results for degree analysis.

**Figure 3:**
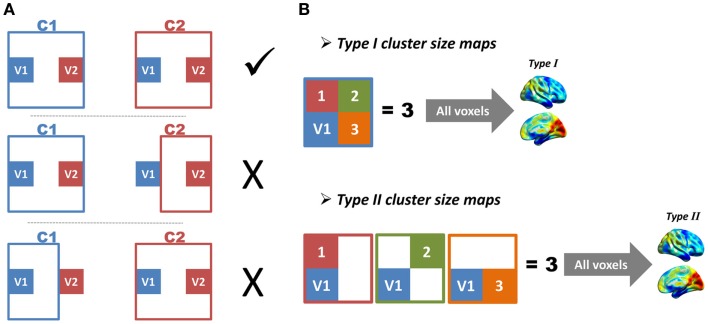
**Illustrations of region-growing error and cluster-size maps are shown**. **(A)** Imagine any two voxels (*V*_1_ and *V*_2_) and their corresponding region-growing clusters (*C*_1_ and *C*_2_). If *C*_1_ includes *V*_2_, *V*_1_ and *V*_2_ will be in the same cluster. In this case, *C*_2_ should also include *V*_1_, otherwise a region-growing error occurs. **(B)** Imagine a voxel *V*_1_, and its corresponding region-growing cluster *C*_1_. We define the total number of voxels in *C*_1_ as *M*_1_, and define total number of other clusters, which includes *V*_1_ as *N*_1_; the *M* and *N* of all voxels constitute type I and type II cluster-size maps, respectively.

As the locations and sizes of functional regions in the human brain were largely unknown, we did not have a golden standard to directly evaluate the performance of the region growing. Two indices were therefore proposed to indirectly evaluate region-growing results. The first index was the region-growing error rate (*RGER*), which can evaluate how many errors the region growing had made. The region-growing error was defined as follows (Figure 3A). Imagine any two voxels (*V*_1_ and *V*_2_) and their corresponding region-growing clusters (*C*_1_ and *C*_2_). If *C*_1_ included *V*_2_, *V*_1_ and *V*_2_ would be in the same cluster. In this case, *C*_2_ should also include *V*_1_, otherwise a region-growing error occurred. The error rate was calculated as the total region-growing errors divided by the total number of pairs of voxels. A low error rate represented a good region growing.

The second index was based on the similarity between two types of cluster-size maps (Figure 3B). Image a voxel *V*_1_ and its corresponding region-growing cluster *C*_1_. The region-growing clusters of other voxels in *C*_1_ would also include *V*_1_, because these voxels were in the same functional region. If the total number of voxels in *C*_1_ was defined as *M*_1_, and the total number of other clusters that included *V*_1_ as *N*_1_, a perfect region growing would require *M*_1_ to be equal to *N*_1_. Based on this criterion, the *M* and *N* of all voxels were calculated, which constituted type I and type II cluster-size maps. These two types of cluster-size maps for each subject were standardized by converting to *z*-scores and were smoothed with an 8 mm FWHM kernel to improve cross-subject overlap. The *z*-score standardization is:

$$z_{i}=\left(x_{i}-\mu \right)∕\sigma ,\;1\leq i\leq N\;$$

where, μ and σ are the mean and SD of the result *x* across all *N* voxels.

Finally, the *ICC* between the two types of maps was calculated across all subjects. A high *ICC* means they are similar, representing a good region growing. The first index (*RGER*) was a global index, whereas the second index was a voxel-wise (local) index for the evaluation of the region growing.

### Degree metrics

A correlation matrix was obtained by computing the Pearson correlation coefficient between any two voxels using their preprocessed time series. Before calculating degree metrics, we needed to define a connectivity threshold (*T_d_*) to determine whether any two voxels were functionally connected. Here, we used the test–retest reliability of degree metrics to find appropriate *T_d_*. If *T_d_* was too low, the test–retest reliability would be compromised because of too many low correlations attributable to signal noise. A higher *T_a_* would increase the test–retest reliability by reducing low correlations. However, when *T_a_* was too high to preserve the real connections, it may reduce the test–retest reliability.

Based on this assumption, a range of connectivity thresholds were tested from 0.15 to 0.5 with a step of 0.05. As shown in the results of step 3, the average correlations of nearest neighbors, after excluding negative and low correlations, were 0.46–0.61 (mean ± SD = 0.52 ± 0.037) and 0.44–0.55 (mean ± SD = 0.49 ± 0.025) for data without and with *GSR*, respectively. Thus, we did not test connectivity thresholds higher than 0.5 because even local strong functional connections may not survive in a very high threshold. We evaluated test–retest reliability as the *ICC* between scan 1 and the average of scans 2 and 3 to improve the estimation of long-term reliability (35, 37, 38).

Four types of degree metrics were computed for a systematical evaluation, including unweighted degree (10), weighted degree, intrinsic connectivity contrast (14), and weighted global brain connectivity (39). Given a certain connectivity threshold *T_a_*, and the correlation between voxels *i* and *j* as *r*(*i, j*) where *i, j* ∈ brain voxels.

Unweighted degree (*U*) is the sum of the number of connections:

$$U\left(i\right)=\sum_{j}f\left(i,\;j\right),\text{where}f\left(i,\;j\right)=\left\{\begin{array}{cc} 1\; & \mathrm{if}r\left(i,j\right)\geq T_{d}\\ 0\; & \mathrm{if}r\left(i,j\right)<T_{d}\\ \; \end{array}\right.$$

Weighted degree (*W*) is the sum of the weights of connections where the weight is the correlation:

$$W\left(i\right)=\sum_{j}w\left(i,\;j\right)\times f\left(i,\;j\right),\text{where}w\left(i,\;j\right)=r\left(i,\;j\right)$$

Intrinsic connectivity contrast (*WS*) is the sum of the weights of connections where the weight is the square of the correlation:

$$WS\left(i\right)=\sum_{j}w_{s}\left(i,\;j\right)\times f\left(i,\;j\right),\text{where}w_{s}\left(i,\;j\right)=r{\left(i,\;j\right)}^{2}$$

We termed the “intrinsic connectivity contrast” as “*WS*” rather than “*ICC-pth*” used in the original paper (14) to avoid confusion with intra-class correlation (*ICC*).

Weighted global brain connectivity (*WF*) is the sum of the weights of connections where the weight is the Fisher’s-*Z*-transformed correlation:

$$\begin{array}{llll} WF\left(i\right) & =\sum_{j}w_{f}\left(i,\;j\right)\times f\left(i,\;j\right),\text{where}\; & & \;\\ w_{f}\left(i,\;j\right) & =\frac{1}{2}\ln\frac{1+r\left(i,\;j\right)}{1-r\left(i,\;j\right)}\; & & \; \end{array}$$

The original formulas of weighted global brain connectivity had no connectivity threshold. Here, we added it to make it comparable with other metrics and termed it as “*WF*” (16, 39). These weightings (*WS* and *WF*) were designed to minimize the influence of weak connections by suppressing weak correlations and emphasizing strong connections.

We then calculated our modified degree metrics using the correction strategy proposed in this study (Figure 1C) to reduce the region-size effect. Based on above summary of previous works, we termed these reduce-size-effect degree metrics as “*U*_RSE_,” “*W*_RSE_,” “*WS*_RSE_,” and “*WF*_RSE_”:

$$\begin{array}{llll} U_{\mathrm{RSE}}\left(i\right) & =\sum_{j}\frac{f\left(i,\;j\right)\times g\left(i,\;j\right)}{s\left(i,\;j\right)}\; & & \;\\ W_{\mathrm{RSE}}\left(i\right) & =\sum_{j}\frac{w\left(i,\;j\right)\times f\left(i,\;j\right)\times g\left(i,\;j\right)}{s\left(i,\;j\right)}\; & & \;\\ WS_{\mathrm{RSE}}\left(i\right) & =\sum_{j}\frac{w_{s}\left(i,\;j\right)\times f\left(i,\;j\right)\times g\left(i,\;j\right)}{s\left(i,\;j\right)}\; & & \;\\ WF_{\mathrm{RSE}}\left(i\right) & =\sum_{j}\frac{w_{f}\left(i,\;j\right)\times f\left(i,\;j\right)\times g\left(i,\;j\right)}{s\left(i,\;j\right)}\; & & \; \end{array}$$

where,

$$g\left(i,\;j\right)=\left\{\begin{array}{cc} 0\; & \mathrm{if}j\in C_{i}\\ 1\; & \mathrm{if}j\notin C_{i}\\ \; \end{array}\right.,\;s\left(i,\;j\right)=\sum_{k}f\left(i,\;k\right),\;k\in C_{j}-C_{i}$$

*C_i_* and *C_j_* are region-growing clusters of voxels *i* and *j*, respectively. *C_j_* − *C_i_* represents set-theoretic difference of *C_j_* in *C_i_*.

The purpose of *g*(*i, j*) was to disregard self-connections between voxels within the same region, and the purpose of *s*(*i, j*) was to regulate connections from voxels of other regions based on the size of those regions (Figure 1C). Take a voxel (*V*_*i*_), for example, when calculating the degree metrics of *V*_*i*_, the connections or the weights between *V*_*i*_ and voxels in the *C*_*i*_ were ignored [the *g*(*i, j*) term], and the connection or the weight between *V*_*i*_ and another connected voxel *V_j_* was divided by the number of voxels of *C_j_* [the *s*(*i, j*) term]. If *C*_*i*_ shared the same elements as *C_j_*, the shared elements were ignored when calculating the number of voxels in *C_j_*. If the correlation between any voxels in *C_j_* and *V*_*i*_ was lower than *T_d_*, these voxels were also ignored when calculating the number of voxels of *C_j_* to avoid overestimation of the cluster size.

### Evaluation of degree metrics

To facilitate group statistics, all degree metrics were standardized by converting to *z*-scores (10), and were smoothed with an 8 mm FWHM kernel to improve cross-subject overlap (7, 12, 16, 20).

Intra-class correlation (ICC) was computed for each degree metric under different thresholds. To evaluate the influence of *T_d_* and to find the optimal threshold for each degree metric, repeated measures ANOVA followed by the Bonferroni multiple comparison test were carried out among each degree metric under different *T_d_*. To evaluate the influence of region-size impact on test–retest reliability, paired two-sample *t*-tests were carried out between the *ICC* of the traditional and modified degree metrics. To evaluate the influence of region-size impact on degree metrics, paired two-sample *t*-tests were carried out between each type of traditional and modified degree metric, and the results were visualized with the BrainNet Viewer^3^.

To investigate the performance of traditional degree metrics and modified degree metrics in statistical group comparisons, two-sample *t*-tests were carried out between the degree metrics of the cocaine-dependent group and those of healthy control group. Statistical *T*-values from clusters that showed significant differences were extracted for a further Wilcoxon matched pairs test between the traditional degree metrics and the modified degree metrics.

## Results

In this study, we proposed a data-driven method to reduce the influence of functional region sizes on degree metrics. A simple example can explain how our method works (Figure 1). Let us assume that the brain contains two large connected functional regions where each consists of two voxels (large regions and system), as well as two small connected functional regions where each consists of one voxel (small regions and system). The degree for all regions is one, as a result of which no hub exists in this network (Figure 1A). In the voxel-wise network, however, the degree of voxels in large regions becomes three whereas the degree of small regions remains one (Figure 1B). A disparity emerges between different levels of hierarchical modular structures where a voxel’s degree scales with the size of its module, and “false-positive” hubs emerge in the large regions and system. We can correct the disparity by (1) disregarding self-connections from voxels within the same region and (2) dividing connections from voxels of other regions by the size of those regions (Figure 1C).

### Adaptive thresholds

The prerequisite of our method is to define functional regions, but the locations and sizes of functional regions in humans are presently unknown. To solve this problem, we relied on the region growing to estimate the region size for each voxel, rather than arbitrarily defined functional regions and systems. To keep the data-driven nature, we considered an adaptive threshold *T_a_* that was used to stop region growing.

**Figure 4:**
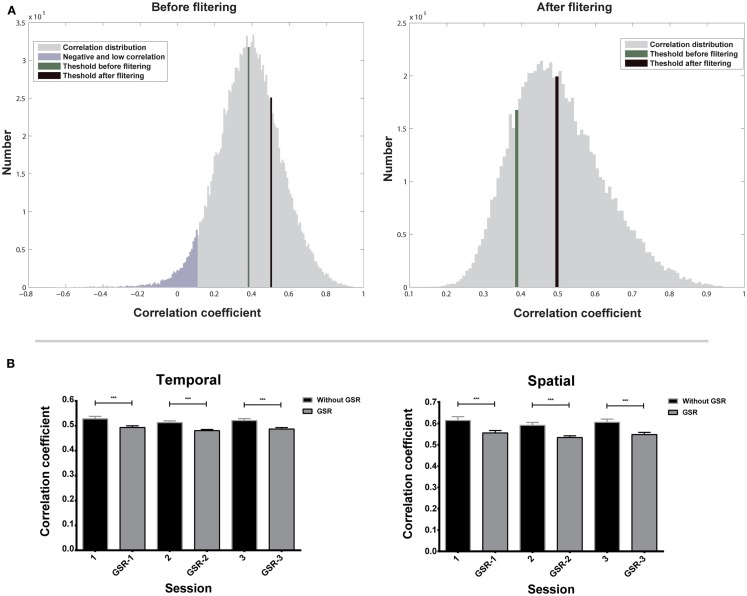
**Evaluation of adaptive thresholds is shown**. **(A)** Correlation distribution of all nearest neighbors to their target voxels before and after two-step filtering. **(B)** Adaptive thresholds based on temporal and spatial correlation of three scans. Bars represent mean ± SEM, ***p < 0.001.

Figure 4A shows the temporal correlation distribution of all nearest neighbors to their target voxels of one subject. The right tail of the distribution contains high correlations from highly homogeneous functional regions. These high correlations should be preserved for the estimation of *T_a_* because they represent the correlations from the same functional regions. On the contrary, the left tail contains negative and low correlations, which should be excluded for the estimation of *T_a_*, because they may represent voxels located near the boundary of functional regions, but not in the same region as the target voxel. The first filtering eliminates all negative and most low correlations (shown in purple in Figure 4A), and the second filtering further reduces the number of low correlations and increases the value of *T_a_*.

Repeated measures ANOVA shows that the effect of scans on *T_a_* is not significant (Figure 4B), for both data without *GSR* [*F*(2,18) = 1.69, *p* = 0.20 for temporal correlation; *F*(2,18) = 1.11, *p* = 0.34 for spatial correlation] and with *GSR* [*F*(2,18) = 2.49, *p* = 0.10 for temporal correlation; *F*(2,18) = 2.47, *p* = 0.10 for spatial correlation]. The result demonstrates that *T_a_* has a good inter-session reliability.

A paired two-sample *t*-test shows that *T_a_* from data without *GSR* is significantly higher than *T_a_* from data with *GSR* (Figure 4B) in both temporal correlation [*t*(56) = 13.17, *p* < 0.0001] and spatial correlation [*t*(56) = 10.11, *p* < 0.0001]. This result indicates that *T_a_* can reflect the *GSR* effect that introduces negative correlations and reduces positive correlations.

### Region growing

**Figure 5:**
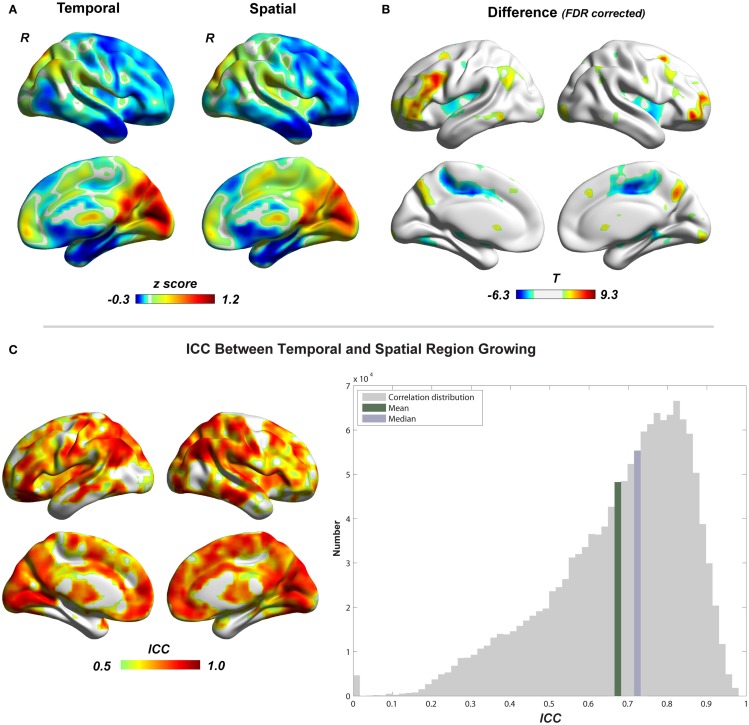
**Comparison between temporal and spatial region growing is shown**. **(A)** Illustrations of temporal and spatial region growing represented by their type I cluster-size maps. **(B)** Paired two-sample *t*-test between temporal and spatial region growing (*p* < 0.05, FDR corrected). **(C)** Similarity between temporal and spatial region growing represented by intra-class correlation.

Figure 5 shows the region-growing results of data without *GSR*, represented by the standardized cluster size of each voxel (type I cluster-size maps). Results with *GSR* were similar. Temporal region growing and spatial region growing produce similar results as revealed by high *ICC* (mean ± SD = 0.69 ± 0.17) between them (Figure 5C). Both results indicate the heterogeneity of functional region sizes. The visual cortex and precuneus/posterior cingulate cortex have prominent larger cluster sizes than the other brain regions. However, temporal and spatial region growing are not identical. Temporal region growing results in significantly larger clusters in the lateral prefrontal cortex, parietal cortex, and precuneus, and smaller clusters in supplementary motor cortex and insular cortex than spatial region growing (Figure 5B, *p* < 0.05, FDR corrected). These differences reflected the nature of temporal and spatial correlation. The information used to calculate temporal correlation is derived from preprocessed time series, whereas the information for spatial correlation involves whole-brain functional connectivity patterns. Voxels located in association brain regions, such as prefrontal cortex, parietal cortex, and cingulate cortex/precuneus, may have similar preprocessed time series (due to real local connections and blurring from data preprocessing), but may have distinct whole-brain functional connectivity patterns, because they are involved in different functional systems. Thus, compared with temporal correlation, the spatial correlation can be a better feature to distinguish voxels in association brain regions, and may result in smaller region-growing clusters in these regions. In contrast, whole-brain functional connectivity patterns of voxels related to sensory and motor function may be less important. The temporal and spatial region-growing results were intersected and resulted in combined region-growing results for degree analysis. The combination of two region growing allows more accurate estimation of functional region sizes because temporal and spatial correlations may reflect different aspects of functional connectivity features.

**Figure 6:**
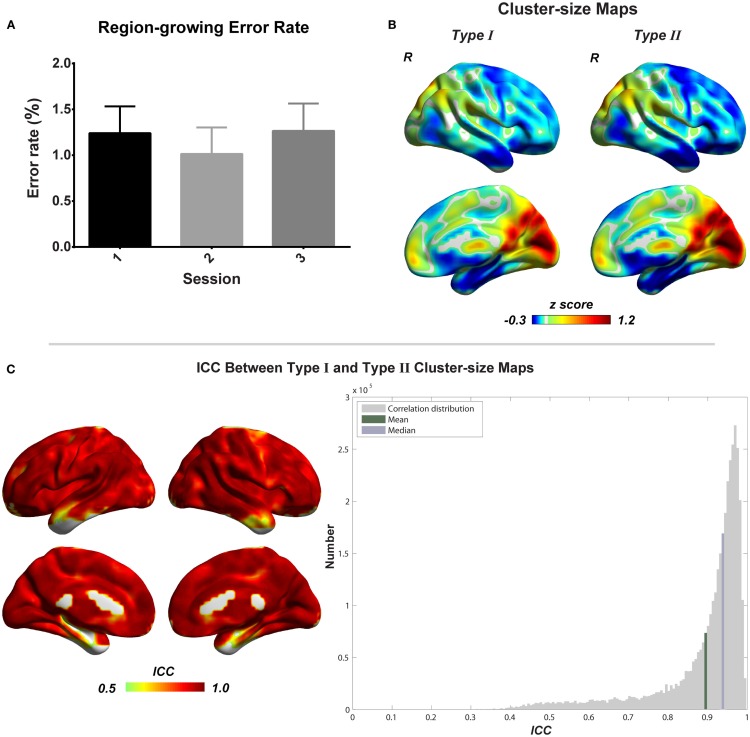
**Evaluation of combined region growing is shown**. **(A)** Region-growing error rates of three scans. Bars represent mean ± SEM. **(B)** Illustrations of type I and type II cluster-size maps. **(C)** The similarity between type I and type II cluster-size maps represented by intra-class correlation.

To evaluate the region-growing results, we also calculated *RGER* and *ICC* between two types of cluster-size maps. As shown in the Figure 6, the *RGER* is low (mean ± SD = 1.17 ± 1.26%), whereas the *ICC* is high (mean ± SD = 0.90 ± 0.12%). Low *RGER* and high *ICC* suggest a good region-growing result.

Although we do not have a golden standard to directly evaluate the performance of the region growing, the region-growing method produces results with low errors and high test–retest reliability and is able to reflect the functional connectivity features of different regions. In addition, the degree results based on the region growing are also consistent with those of Power et al. (22) that explored different methods (See Discussion). Thus, the region-growing method achieves our expectation.

### Degree metrics

Before calculating degree metrics, we needed to define a connectivity threshold (*T_d_*) to determine whether two voxels were functionally connected. We first examined the influence of *T_d_* on the test–retest reliability of degree metrics.

**Figure 7:**
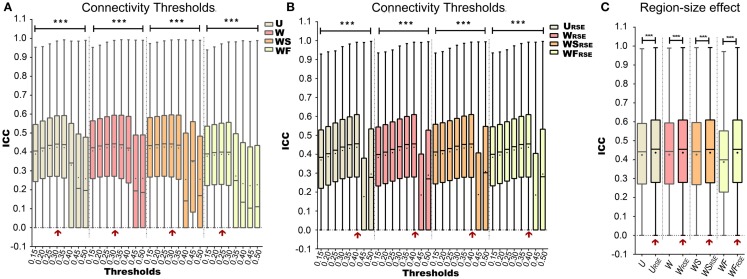
**Test–retest reliability of degree metrics is shown**. Test–retest reliability of **(A)** traditional degree metrics and **(B)** modified degree metrics under different connectivity thresholds. **(C)** Comparison between the test–retest reliability of traditional and modified degree metrics. The entry with highest test–retest reliability is marked with a red arrow. Box plots represent the median, the upper and lower quartiles, and the minimum and maximum data values; black dots represent the mean data values, ***p < 0.001.

Figures 7A,B show how *T_d_* influences the test–retest reliability of degree metrics from data without *GSR*. The results from data with *GSR* are similar. Regardless of the type of degree metrics, higher *T_d_* always results in higher *ICC* until it reaches an optimal. *ICC* is dramatically reduced when *T_d_* exceeds the optimal threshold. This result supports the assumption that higher *T_d_* reduces low correlations (mostly attributed to noise) and improves the test–retest reliability, but that when *T_d_* is too high, it may compromise real network connections and reduce the test–retest reliability.

After defining the optimal *T_d_*, we evaluated the region-size impact on the test–retest reliability of the degree metrics (Figure 7C; Table 1). Modified degree metrics have significantly higher *ICC* than traditional degree metrics for all four types in both data without *GSR* and data with *GSR*. Thus, region-size impact compromised the test–retest reliability of degree metrics. If test–retest reliability of degree metrics is regarded as the criterion for comparing different methods, our modified method outperforms the traditional method.

**Table 1:** **Paired two-sample *t*-test between the ICC of modified degree metrics and that of traditional degree metrics in the NY_TRT dataset**.

	Without GSR	With GSR
*U*_RSE_ − *U*	*t*(23424) = 27.50, *p* < 0.0001	*t*(23424) = 2.26, *p* < 0.05
*W*_RSE_ − *W*	*t*(23424) = 26.14, *p* < 0.0001	*t*(23424) = 3.33, *p* < 0.001
*WS*_RSE_ − *WS*	*t*(23424) = 26.12, *p* < 0.0001	*t*(23424) = 4.25, *p* < 0.0001
*WF*_RSE_ − *WF*	*t*(23424) = 65.49, *p* < 0.0001	*t*(23424) = 46.14, *p* < 0.0001

**Figure 8:**
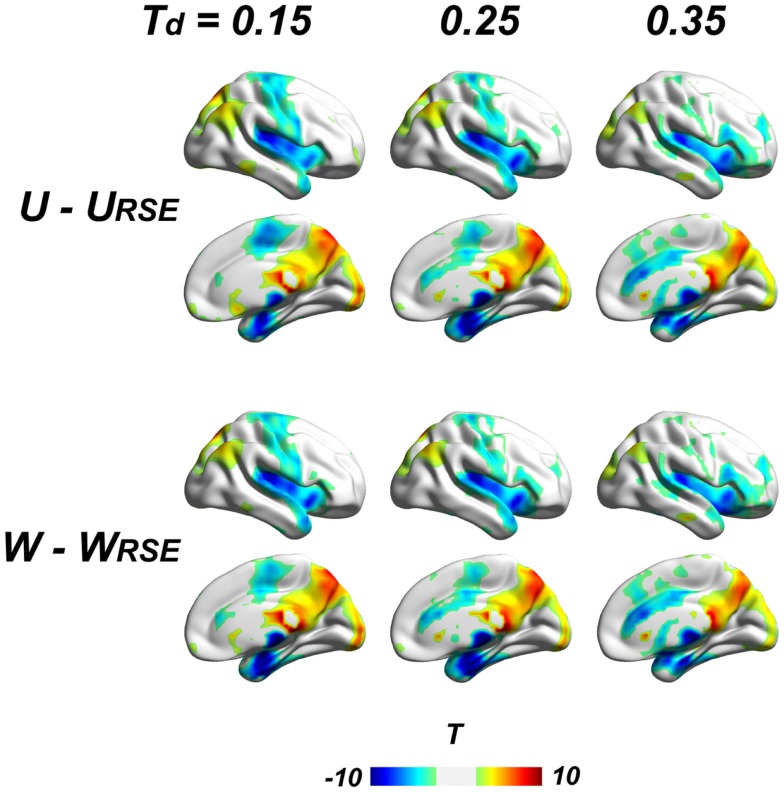
**Comparison between traditional and modified degree metrics is shown**. Results of the unweighted (*U* − *U*_RSE_) and weighted degree metrics (*W*  − *W*_RSE_) are shown (*p* < 0.05, FDR corrected). Results of derivative metrics (*WS* − *WS*_RSE_ and *WF* − *WF*_RSE_) are similar as the displayed results. The displayed results are based on data without global signal regression; the results with other data-preprocessing procedures are presented in the Figure S1 in Supplementary Material.

To further examine the impact of region sizes, we compared traditional degree metrics (*U, W, WS*, and *WF*) with modified degree metrics (*U*_RSE_, *W*_RSE_, *WS*_RSE_, and *WF*_RSE_) under different connectivity thresholds by paired two-sample *t*-test. We conducted the comparison at a range of connectivity thresholds with *T_d_* ≤ 0.45, because very high *T_a_* results in low test–retest reliability of degree metrics and may comprise the network architecture (Figure 7). All types of degree metrics produce similar results (Figure 8), even under different data pre-processing procedures (Figure S1 in Supplementary Material). Compared with the modified degree metrics, the traditional degree metrics have a significantly higher degree in the visual cortex, precuneus/posterior cingulate cortex, posterior parietal cortex, and a significantly lower degree in the insular cortex, sensory and motor cortex, supplementary motor area, anterior cingulate cortex, parahippocampus, and areas around temporal pole (*p* < 0.05, FDR corrected). This result is consistent with region-growing results where larger brain regions tend to have higher degree (Figures 6B and 8).

**Figure 9:**
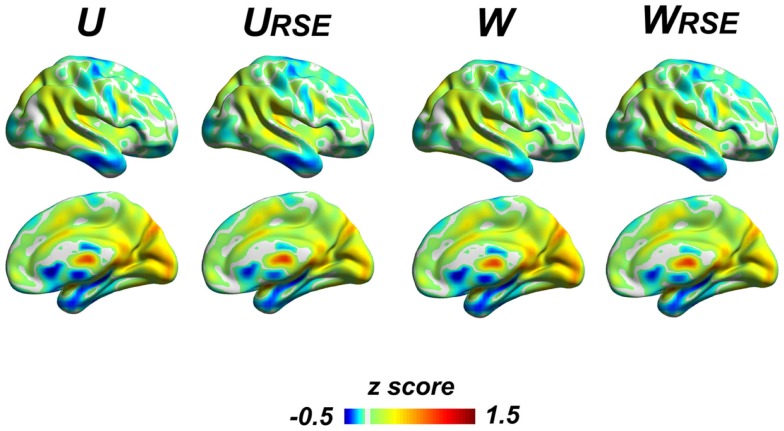
**The overall degree distribution is similar across different types of degree metrics**. The unweighted and weighted degree metrics (*U, U*_RSE_, *W*, and *W*_RSE_) are displayed; other degree metrics (*WS, WS*_RSE_*, WF, WF*_RSE_) are presented in the Figure S2 in Supplementary Material. Brain regions with high degree are consistently observed in the visual cortex, precuneus/posterior cingulate cortex, thalamus, posterior parietal cortex, anterior cingulate cortex, anterior insular cortex, and medial temporal cortex.

Although the region-size variance has a significant effect on degree metrics, the effect does not affect the distribution of high-degree regions (Figure 9), even under very high connectivity thresholds that may induce low test–retest reliability (Figure S2 in Supplementary Material). All degree metrics produce similar patterns of degree distribution. Brain regions with a high degree are consistently observed in the visual cortex, precuneus/posterior cingulate cortex, thalamus, posterior parietal cortex, anterior cingulate cortex, anterior insular cortex, and medial temporal cortex.

**Figure 10:**
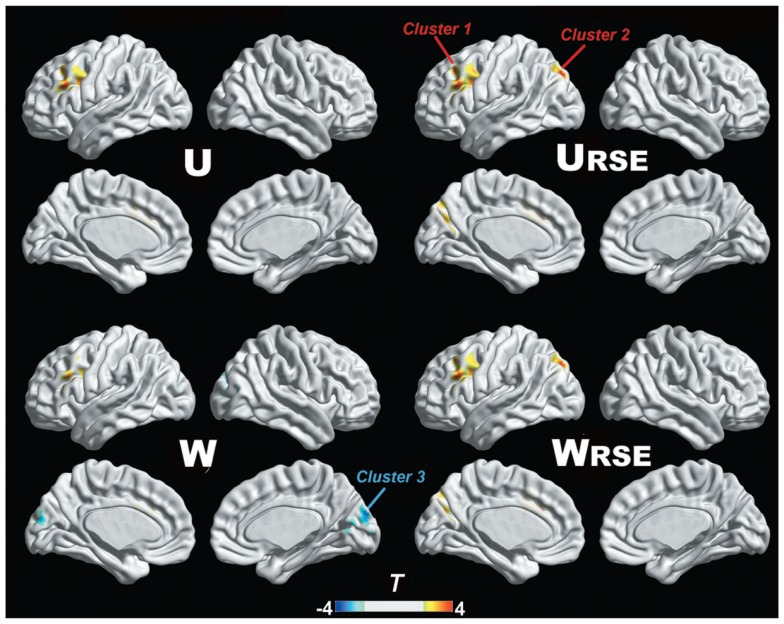
**Degree comparison between the cocaine-dependent group and the control group is shown**. The left lateral prefrontal cortex (cluster 1), left posterior parietal cortex (cluster 2), and visual cortex (cluster 3) show significant differences in degree metrics between the two groups (*p* < 0.05, FDR corrected).

**Figure 11:**
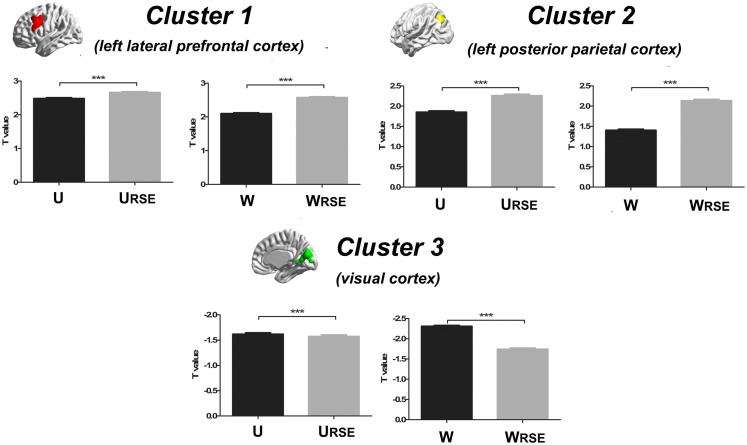
**Statistical *T*-values comparison between the traditional degree metrics and the modified degree metrics is shown**. Cluster 1 is the left lateral prefrontal cortex (true-positive), cluster 2 is the left posterior parietal cortex (true-positive), and cluster 3 is the visual cortex (false-positive). Bars represent mean ± SEM, ***p < 0.001.

In addition, we compared the performance of the traditional degree metrics and the modified degree metrics in the statistical comparison between the cocaine-dependent and control group. Figure 10 shows results of two-sample *t*-tests between the degree metrics of the cocaine-dependent group and those of the control group, under connectivity thresholds 0.2. Results under the other thresholds are similar as that under 0.2. When the traditional degree metrics are used, the cocaine-dependent group has a significantly higher unweighted degree (*U*) in the left lateral prefrontal cortex and a lower weighted degree (*W*) in the visual cortex than the control group (*p* < 0.05, corrected). When the modified degree metrics are used, the cocaine-dependent group has a significantly higher unweighted (*U*_RSE_) and weighted degree (*W*_RSE_) in the left lateral prefrontal cortex and left posterior parietal cortex than the control group. Thus, the left lateral prefrontal cortex (cluster 1), left posterior parietal cortex (cluster 2), and visual cortex (cluster 3) show significant differences in degree between the two groups. Statistical *T*-values from voxels in these clusters were extracted, and Wilcoxon matched pairs tests were carried out between the traditional degree metrics and the modified degree metrics for each cluster. As shown in Figure 11, the modified degree metrics produce significantly higher *T*-values in the left lateral prefrontal cortex and the left posterior parietal cortex (true-positive), but lower *T*-values in the visual cortex (false-positive, see Discussion) than the traditional degree metrics.

## Discussion

Previous studies proposed several degree metrics to search for the hubs in the voxel-wise functional brain network from the basic unweighted (*U*) and weighted degree (*W*) to their derivative metrics (for example *WS* and *WF*) that were designed to minimize the influence of weak connections and emphasize strong connections (10, 14, 39). Although all of them showed their capability of locating hubs (Figure 9), these traditional degree metrics were significantly affected by the functional region sizes. Consistent with the findings of Power et al. (22), we found that the large brain regions/systems had the overestimated importance in the brain network when using these traditional degree metrics. However, our results showed that the impact did not change the locations of hubs, whereas Power et al. (22) discovered new hubs by using participate coefficient and community density. These two findings were not actually contradictory but complementary, because degree metrics and participate coefficient/community density of the Power et al. (22) revealed different perspectives of network properties, the former emphasizing the number of connections (both within-module and between-module connections) and the latter emphasizing participation in multiple sub-networks (between-module connections). In addition, both studies agreed that the degree of default mode network and visual cortex were overestimated by traditional degree metrics. The set of newly discovered “hubs” in Power et al. (22) were also the same regions, which showed underestimated degree by the traditional degree metrics in our evaluation, including the insular cortex, anterior cingulate cortex, and anterior frontal cortex under most connectivity thresholds.

Compared with the traditional metrics, our modified metrics significantly reduced the impact of the region size and improved the test–retest reliability. Furthermore, we compared different degree metrics between the cocaine-dependent group and the control group to evaluate the performance of these metrics in group-statistic comparisons. The modified degree metrics showed a higher significant difference in the left lateral prefrontal cortex and left posterior parietal cortex, and a lower significant difference in the visual cortex than the traditional degree metrics (Figure 11). The lateral prefrontal cortex and posterior parietal cortex were important nodes in the dorsal attention network, which is associated with cocaine addiction (28, 40–42). Using the same dataset, a previous study also demonstrated the impaired connectivity of the lateral prefrontal cortices and the posterior parietal areas in cocaine-dependent group (28). In contrast, no evidence supported abnormal functional connectivity of the visual cortex in chronic cocaine addiction. The significant false-positive difference in the traditional degree in the visual cortex may be caused by the impact arising from its large functional region size. With modified degree metrics, the statistical significance between the cocaine-dependent group and the control group in the visual cortex (false-positive) decreased, and statistical significance in the lateral prefrontal cortex and posterior parietal areas (true-positive) increased. These results suggest that region size may affect the degree comparison and the modified degree metrics can reduce the impact to improve the sensitivity of degree metrics in group-statistic comparisons.

Other degree-based methods were also proposed in previous studies, which may be beneficial in reducing the impact of region size. These methods were achieved either by excluding short-distance connections or by emphasizing long-distance connections (15, 43). However, a voxel’s degree scales not only the size of the functional region to which it belongs (short-distance contributions) but also the sizes of functional regions to which it connects (mostly long-distance contributions). Therefore, these previous methods can only help to reduce the impact from the first term but not the second term, whereas our modified method can reduce both terms. In conclusion, we believe that our modified method will have wide applications, as it inherits the data-driven merit of the degree-based method, overcomes the drawback from the region-size impact, improves the test–retest reliability, and enhances the sensitivity of degree metrics in group-statistic comparisons.

## Conflict of Interest Statement

The authors declare that the research was conducted in the absence of any commercial or financial relationships that could be construed as a potential conflict of interest.

## Supplementary Material

The Supplementary Material for this article can be found online at http://www.frontiersin.org/Journal/10.3389/fneur.2014.00199/abstract

## Footnotes

^1^http://www.nitrc.org/projects/nyu_trt/

^2^http://www.fil.ion.ucl.ac.uk/spm

^3^http://www.nitrc.org/projects/bnv/

## References

[1] Bullmore E Sporns O The economy of brain network organization. Nat Rev Neurosci (2012) 13:336–49 10.1038/nrn3214 22498897

[2] Felleman DJ van Essen DC Distributed hierarchical processing in the primate cerebral cortex. Cereb Cortex (1991) 1:1–47 10.1093/cercor/1.1.1 1822724

[3] Scannell JW Burns GA Hilgetag CC O’Neil MA Young MP The connectional organization of the cortico-thalamic system of the cat. Cereb Cortex (1999) 9:277–99 10.1093/cercor/9.3.277 10355908

[4] Sporns O Honey CJ Kotter R Identification and classification of hubs in brain networks. PLoS One (2007) 2:e1049 10.1371/journal.pone.0001049 17940613 PMC2013941

[5] Song M Jiang TZ A review of functional magnetic resonance imaging for brainnetome. Neurosci Bull (2012) 28:389–98 10.1007/s12264-012-1244-4 22833037 PMC5560259

[6] Zuo NM Cheng J Jiang TZ Diffusion magnetic resonance imaging for brainnetome: a critical review. Neurosci Bull (2012) 28:375–88 10.1007/s12264-012-1245-3 22833036 PMC5560260

[7] van den Heuvel MP Stam CJ Boersma M Hulshoff Pol HE Small-world and scale-free organization of voxel-based resting-state functional connectivity in the human brain. Neuroimage (2008) 43:528–39 10.1016/j.neuroimage.2008.08.010 18786642

[8] Biswal B Yetkin FZ Haughton VM Hyde JS Functional connectivity in the motor cortex of resting human brain using echo-planar MRI. Magn Reson Med (1995) 34:537–41 10.1002/mrm.1910340409 8524021

[9] Wasserman S Social Network Analysis: Methods and Applications. Cambridge: University Press (1994).

[10] Buckner RL Sepulcre J Talukdar T Krienen FM Liu H Hedden T Cortical hubs revealed by intrinsic functional connectivity: mapping, assessment of stability, and relation to Alzheimer’s disease. J Neurosci (2009) 29:1860–73 10.1523/JNEUROSCI.5062-08.2009 19211893 PMC2750039

[11] Murphy K Birn RM Handwerker DA Jones TB Bandettini PA The impact of global signal regression on resting state correlations: are anti-correlated networks introduced? Neuroimage (2009) 44:893–905 10.1016/j.neuroimage.2008.09.036 18976716 PMC2750906

[12] Tomasi D Volkow ND Functional connectivity density mapping. Proc Natl Acad Sci U S A (2010) 107:9885–90 10.1073/pnas.1001414107 20457896 PMC2906909

[13] Fransson P Aden U Blennow M Lagercrantz H The functional architecture of the infant brain as revealed by resting-state fMRI. Cereb Cortex (2011) 21:145–54 10.1093/cercor/bhq071 20421249

[14] Martuzzi R Ramani R Qiu ML Shen XL Papademetris X Constable RT A whole-brain voxel based measure of intrinsic connectivity contrast reveals local changes in tissue connectivity with anesthetic without a priori assumptions on thresholds or regions of interest. Neuroimage (2011) 58:1044–50 10.1016/j.neuroimage.2011.06.075 21763437 PMC3183817

[15] Tomasi D Volkow ND Functional connectivity hubs in the human brain. Neuroimage (2011) 57:908–17 10.1016/j.neuroimage.2011.05.024 21609769 PMC3129362

[16] Scheinost D Benjamin J Lacadie CM Vohr B Schneider KC Ment LR The intrinsic connectivity distribution: a novel contrast measure reflecting voxel level functional connectivity. Neuroimage (2012) 62:1510–9 10.1016/j.neuroimage.2012.05.073 22659477 PMC3538880

[17] Tomasi D Volkow ND Gender differences in brain functional connectivity density. Hum Brain Mapp (2012) 33:849–60 10.1002/hbm.21252 21425398 PMC3250567

[18] Tomasi D Volkow ND Laterality patterns of brain functional connectivity: gender effects. Cereb Cortex (2012) 22:1455–62 10.1093/cercor/bhr230 21878483 PMC3450858

[19] Tomasi D Volkow ND Resting functional connectivity of language networks: characterization and reproducibility. Mol Psychiatry (2012) 17:841–54 10.1038/mp.2011.177 22212597 PMC3323720

[20] Zuo XN Ehmke R Mennes M Imperati D Castellanos FX Sporns O Network centrality in the human functional connectome. Cereb Cortex (2012) 22:1862–75 10.1093/cercor/bhr269 21968567

[21] Power JD Fair DA Schlaggar BL Petersen SE The development of human functional brain networks. Neuron (2010) 67:735–48 10.1016/j.neuron.2010.08.017 20826306 PMC2941973

[22] Power JD Schlaggar BL Lessov-Schlaggar CN Petersen SE Evidence for hubs in human functional brain networks. Neuron (2013) 79:798–813 10.1016/j.neuron.2013.07.035 23972601 PMC3838673

[23] Wang J Wang L Zang Y Yang H Tang H Gong Q Parcellation-dependent small-world brain functional networks: a resting-state fMRI study. Hum Brain Mapp (2009) 30:1511–23 10.1002/hbm.20623 18649353 PMC6870680

[24] Zalesky A Fornito A Harding IH Cocchi L Yucel M Pantelis C Whole-brain anatomical networks: does the choice of nodes matter? Neuroimage (2010) 50:970–83 10.1016/j.neuroimage.2009.12.027 20035887

[25] Fornito A Zalesky A Breakspear M Graph analysis of the human connectome: promise, progress, and pitfalls. Neuroimage (2013) 80:426–44 10.1016/j.neuroimage.2013.04.087 23643999

[26] Tian X Liu C Jiang T Rizak J Ma Y Hu X Feature-reduction and semi-simulated data in functional connectivity-based cortical parcellation. Neurosci Bull (2013) 29:333–47 10.1007/s12264-013-1339-6 23700282 PMC5561846

[27] Biswal BB Mennes M Zuo XN Gohel S Kelly C Smith SM Toward discovery science of human brain function. Proc Natl Acad Sci U S A (2010) 107:4734–9 10.1073/pnas.0911855107 20176931 PMC2842060

[28] Kelly C Zuo XN Gotimer K Cox CL Lynch L Brock D Reduced interhemispheric resting state functional connectivity in cocaine addiction. Biol Psychiatry (2011) 69:684–92 10.1016/j.biopsych.2010.11.022 21251646 PMC3056937

[29] Yan CG Zang YF DPARSF: a MATLAB toolbox for “pipeline” data analysis of resting-state fMRI. Front Syst Neurosci (2010) 4:13 10.3389/fnsys.2010.00013 20577591 PMC2889691

[30] Song XW Dong ZY Long XY Li SF Zuo XN Zhu CZ REST: a toolkit for resting-state functional magnetic resonance imaging data processing. PLoS One (2011) 6:e25031 10.1371/journal.pone.0025031 21949842 PMC3176805

[31] Ashburner J Friston KJ Unified segmentation. Neuroimage (2005) 26:839–51 10.1016/j.neuroimage.2005.02.018 15955494

[32] Ashburner J A fast diffeomorphic image registration algorithm. Neuroimage (2007) 38:95–113 10.1016/j.neuroimage.2007.07.007 17761438

[33] Weissenbacher A Kasess C Gerstl F Lanzenberger R Moser E Windischberger C Correlations and anticorrelations in resting-state functional connectivity MRI: a quantitative comparison of preprocessing strategies. Neuroimage (2009) 47:1408–16 10.1016/j.neuroimage.2009.05.005 19442749

[34] Power JD Barnes KA Snyder AZ Schlaggar BL Petersen SE Spurious but systematic correlations in functional connectivity MRI networks arise from subject motion. Neuroimage (2012) 59:2142–54 10.1016/j.neuroimage.2011.10.018 22019881 PMC3254728

[35] Yan CG Cheung B Kelly C Colcombe S Craddock RC Di Martino A A comprehensive assessment of regional variation in the impact of head micromovements on functional connectomics. Neuroimage (2013) 76:183–201 10.1016/j.neuroimage.2013.03.004 23499792 PMC3896129

[36] Chen G Saad ZS Britton JC Pine DS Cox RW Linear mixed-effects modeling approach to fMRI group analysis. Neuroimage (2013) 73:176–90 10.1016/j.neuroimage.2013.01.047 23376789 PMC3638840

[37] Shehzad Z Kelly AM Reiss PT Gee DG Gotimer K Uddin LQ The resting brain: unconstrained yet reliable. Cereb Cortex (2009) 19:2209–29 10.1093/cercor/bhn256 19221144 PMC3896030

[38] Zuo XN Di Martino A Kelly C Shehzad ZE Gee DG Klein DF The oscillating brain: complex and reliable. Neuroimage (2010) 49:1432–45 10.1016/j.neuroimage.2009.09.037 19782143 PMC2856476

[39] Cole MW Pathak S Schneider W Identifying the brain’s most globally connected regions. Neuroimage (2010) 49:3132–48 10.1016/j.neuroimage.2009.11.001 19909818

[40] Dosenbach NU Fair DA Miezin FM Cohen AL Wenger KK Dosenbach RA Distinct brain networks for adaptive and stable task control in humans. Proc Natl Acad Sci U S A (2007) 104:11073–8 10.1073/pnas.0704320104 17576922 PMC1904171

[41] Dosenbach NU Fair DA Cohen AL Schlaggar BL Petersen SE A dual-networks architecture of top-down control. Trends Cogn Sci (2008) 12:99–105 10.1016/j.tics.2008.01.001 18262825 PMC3632449

[42] Smith SM Fox PT Miller KL Glahn DC Fox PM Mackay CE Correspondence of the brain’s functional architecture during activation and rest. Proc Natl Acad Sci U S A (2009) 106:13040–5 10.1073/pnas.0905267106 19620724 PMC2722273

[43] Power JD Cohen AL Nelson SM Wig GS Barnes KA Church JA Functional network organization of the human brain. Neuron (2011) 72:665–78 10.1016/j.neuron.2011.09.006 22099467 PMC3222858

