# Supplementary figures and images for: A Data-Driven Method to Reduce the Impact of Region Size on Degree Metrics in Voxel-Wise Functional Brain Networks
Source: Front Neurol. 2014 Oct 13;5:199. doi: 10.3389/fneur.2014.00199 (PMC4195315; doi:10.3389/fneur.2014.00199)

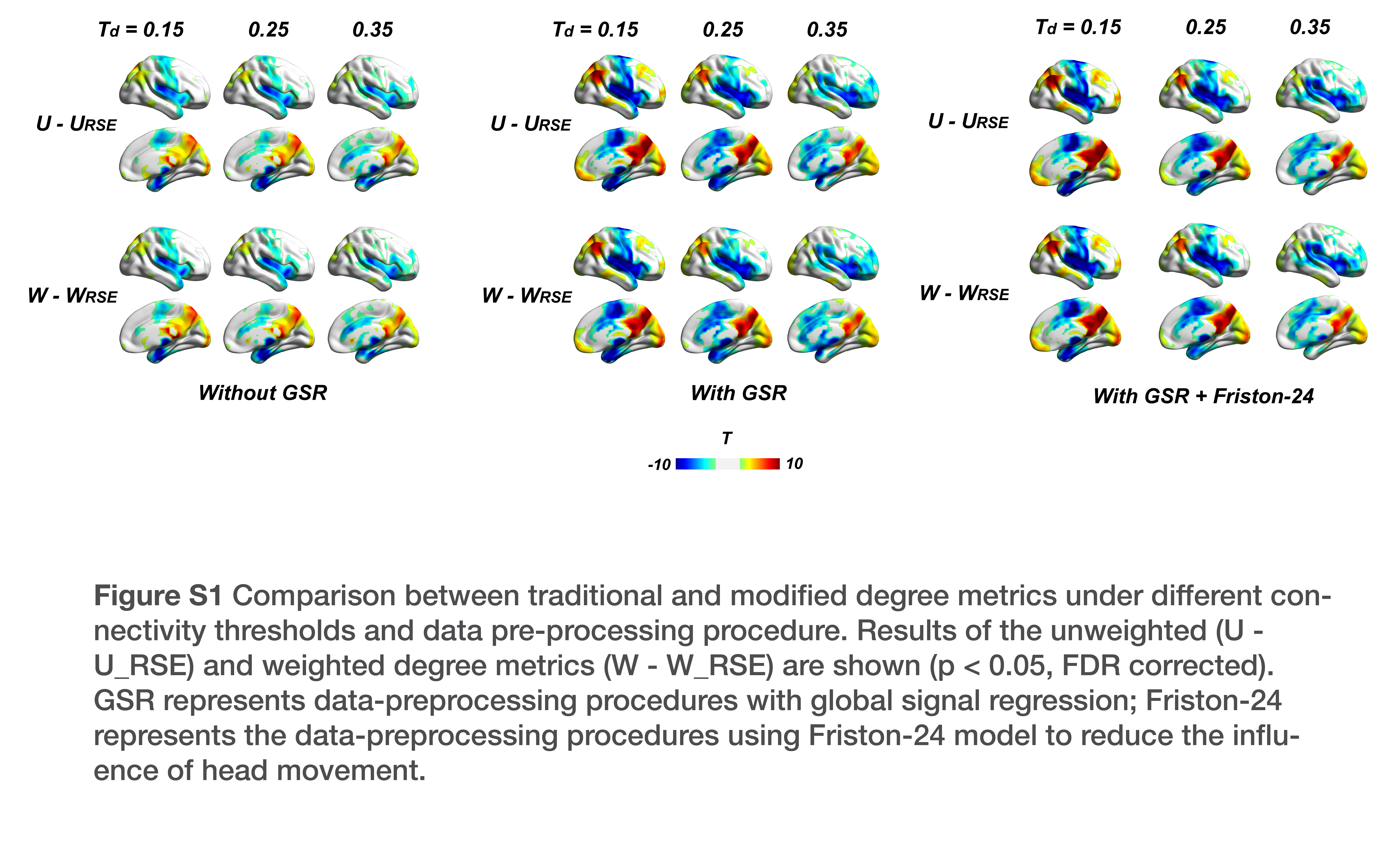

Supplement: Supplementary file 1 [file Image1.TIF]

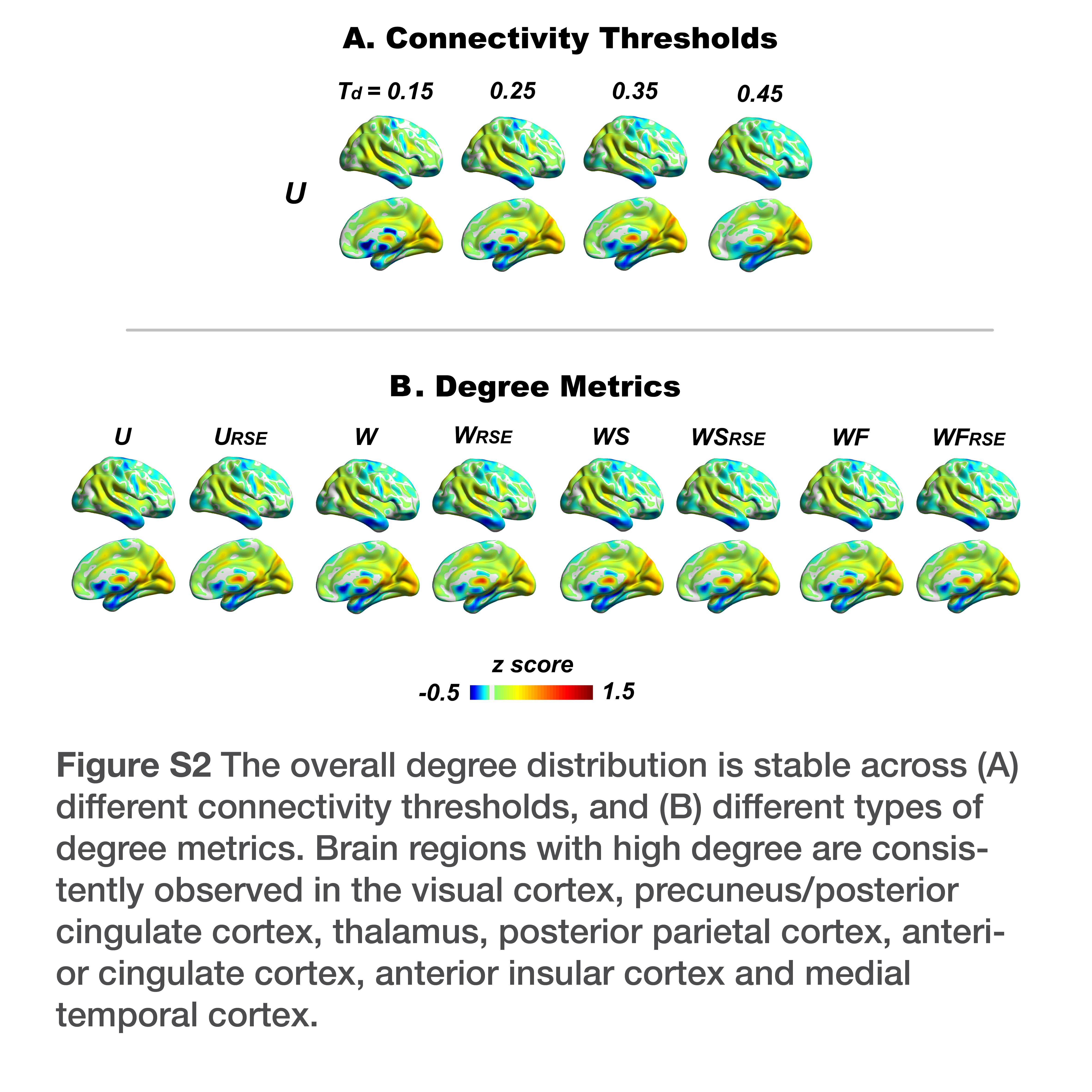

Supplement: Supplementary file 2 [file Image2.TIF]
